# The Evolution of Scientific Knowledge in Childhood Asthma over Time: A Surprising History

**DOI:** 10.3390/children11020262

**Published:** 2024-02-18

**Authors:** Laura Venditto, Sonia Morano, Giuliana Ferrante, Michele Piazza, Laura Tenero, Giorgio Piacentini, Luca Pecoraro

**Affiliations:** Pediatric Clinic, Department of Surgical Sciences, Dentistry, Gynecology and Pediatrics, University of Verona, 37126 Verona, Italy; laura.venditto@studenti.univr.it (L.V.); sonia.morano@studenti.univr.it (S.M.); michele.piazza@univr.it (M.P.);

**Keywords:** severe asthma, biologics, childhood asthma

## Abstract

Asthma is a disease that has been described since the times of Hammurabi. However, it is only since the 1960s that effective therapeutic strategies have been available. Pathogenic mechanisms underlying the disease have been deeply studied, contributing to creating a “patient-specific asthma” definition. Biological drugs have been approved over the last twenty years, improving disease management in patients with severe asthma via a “precision medicine-driven approach”. This article aims to describe the evolution of scientific knowledge in childhood asthma, focusing on the most recent biological therapies and their indications for patients with severe asthma.

## 1. Introduction

Asthma is the most frequent chronic inflammatory disease of childhood. Asthma is defined by reversible bronchoconstriction, thickening of the airway walls, and increased mucus production, resulting in variable expiratory airflow limitation. Typically, children with asthma show symptoms like shortness of breath, chest tightness, wheezing and cough, often triggered by exercise, allergen/irritant exposure, viral infections, and weather changes [1]. Since 1960, effective therapeutic strategies have been available. Moreover, pathogenic mechanisms underlying this disease have been deeply studied, contributing to the development of biological drugs with a significant improvement in disease management of severe asthma. This review aims to describe the evolution of scientific knowledge in childhood asthma, focusing on the most recent biological therapies and their indications for patients with severe asthma.

## 2. Asthma: An Ancient Disease

Knowledge about asthma and its treatment has dramatically evolved over time (Table 1). The first finding of a pathology that recalled asthma was in 1754 BCE in the Code of Hammurabi, where it was reported as “breathlessness”. The term “asthma” was coined by Hippocrates of Kos (460-370 BCE) and appeared for the first time in Ancient Greek as “aazein”. For a long time, the treatment of asthma consisted of rest, a dry environment, proper hygiene, special diets, red wine, and the avoidance of opium. Black coffee and thorn apple plant-derived belladonna alkaloids have played a role in the history of the treatment of asthma, too. The first primitive inhaler was developed by John Mudge only in the 18th century, while albuterol and inhaled beclomethasone were developed in 1967 and 1972 [2]. Since 1960, thanks to the discovery of these two drugs, the advances in asthma management have been exponential. Specifically, a paradigm shift was seen, shifting the focus from the impairment related to asthma to its potential risks. Asthma knowledge and management are now focused on asthma control, bringing attention to the disease progression and assessing the risks for future exacerbations and the adverse effects caused by medications [3].

## 3. The New Concept of Different Types of Asthma

The evolution in asthma knowledge and management has consequently changed the definition of asthma compared to the past. Historically, asthma was understood as a unique disease requiring the same treatment in all patients. Nowadays, asthma is considered to have different pathophysiological underlying mechanisms, even though they lead to a common clinical presentation. These pathophysiological features contribute to creating a patient-specific asthma definition based on symptom frequency, medication use, the presence of comorbidities, atopic status, and pulmonary function testing [4]. Consequently, a personalized therapeutic approach must be required for each specific patient. Also, asthma therapy has evolved over time. International guidelines, mainly defined by the National Asthma Education and Prevention Program (NAEPP), the European Respiratory Society (ERS), the Global Initiative for Asthma (GINA), and the National Institute for Health and Care Excellence (NICE), provide a step-by-step algorithm to guide the decision path to achieve disease control. The first step is represented by as-needed albuterol, and as-needed or daily low-dose inhaled corticosteroids (ICS). The next steps include increasing ICS dose and adding further medications until control is achieved [1,3]. The year 2005 represented a turning point due to the official recommendation by the European Medicines Agency (EMA) of the first biologic drug, omalizumab, to treat severe asthma in Europe.

In childhood severe asthma, two major endotypes have been described: T2-high asthma (allergic/non-allergic) and T2-low asthma (neutrophilic/paucigranulocytic) [5]. T2-high allergic asthma is the most frequent type in the pediatric population, driven by type 2 inflammation cytokines, such as IL-4, IL-5, IL-9 and IL-13 [6]. Airway exposure to the allergen activates the production of some epithelial-derived cytokines, such as thymic stromal lymphopoietin (TSLP), IL-33 and IL-25, causing the differentiation of lymphoid progenitors into innate type 2 lymphocytes (ILC2) and Th2 lymphocytes able to release mediators that feed and perpetuate inflammation. [6]. IL-4 is involved in the production of IgE, while IL-5 is implicated in the proliferation and differentiation of eosinophils, and IL-13 promotes tissue remodeling of the bronchi. The inflammation mediated by these cytokines, the metaplasia of mucus cells and the increased mucus production contribute to the hyperreactivity of the airways in asthmatic patients [7]. Eosinophils have a key role in T2-high asthma [8]. Specifically, blood eosinophil count is considered a good surrogate for airway eosinophilic inflammation [9]. On the other hand, T2-low asthma is defined by the presence of neutrophils in sputum or by the absence (or normal levels) of eosinophils or by other T2 markers in sputum, airway biopsies or blood [10]. In the pathogenic pathway T helper 1 (Th1) and T helper 17 (Th17) cells and neutrophils are involved with the production of cytokines such as IL-1ß, IL-6, IL-8, IL-17A/F, IFN-γ, and TNF-α [10]. The coexistence of both cells from T2-high and T2-low asthma could develop a mixed endotype [11].

## 4. Different Endotypes for Different Therapies in Severe Asthma

The classification of asthma endotypes is fundamental to guide a targeted therapeutic approach, especially for problematic severe asthma, defined as uncontrolled asthma despite an adequate treatment with a high dose of ICS and long-acting beta2-agonist or that requires a high dose of ICS-LABA to remain controlled. The estimated prevalence of severe asthma is around 2.1–10% among children with asthma [12]. Children with severe asthma can be affected by persistent symptoms, life-threatening acute attacks, neuropsychological consequences, and side effects related to the use of high-dose oral corticosteroids (OCS) [13]. In most cases, severe childhood asthma is characterized by an early onset and multiple aeroallergens sensitization, elevated total serum immunoglobulin E (IgE) levels, and high blood eosinophil count [13]. Actually, more than one biological option is available for the treatment of children with severe T2-high asthma, while since 2022, a biological therapy for children with severe T2-low asthma has been available: tezepelumab. However, limited treatment options have been approved for pediatric age, such as omalizumab, mepolizumab, dupilumab, benralizumab, and tezepelumab (Table 2) [14,15].

## 5. Omalizumab

Omalizumab is the first biologic drug approved for asthma and currently approved for children aged ≥6 years. It is an anti-IgE monoclonal antibody administered via subcutaneous injection every 2–4 weeks. The dose is based on total serum IgE and body weight within a limit of the dosage established by a specific nomogram [14]. There is insufficient evidence to administer a dose outside this nomogram, especially for younger, overweight and obese children [16]. The total doses range from 75 to 600 mg (0.016 mg/kg/IgE (I.U./mL) per 4 weeks. In fact, the maximum possible dose is 600 mg every 2 weeks in Europe and 375 mg every 2 weeks in the USA. Plasma half-life time is about 26 days. Moreover, 75 mg and 150 mg prefilled syringes and 150 mg ampoules are currently available, with the possibility of being administered at home [17]. The omalizumab’s target is the Fc region of free serum IgE, preventing its binding to FcεR1 receptors on mast cells and basophils, reducing free IgE and inducing the down-regulation of their receptors. Omalizumab is recommended as an additional therapy for children affected by severe asthma sensitized to perennial inhalant allergens (such as animal dander, dust mites, cockroaches or molds). The criteria for the prescription require a specific range of serum IgE and of body weight in addition to a specific number of exacerbations over the last year [14,16,18,19,20]. As well as for asthma, omalizumab is also approved for nasal polyps and chronic spontaneous (idiopathic) urticaria [14]. Deepening the efficacy of omalizumab, some studies demonstrated a significant reduction in severe asthma exacerbation, OCS administration and hospitalization in patients affected by severe asthma. Thanks to these effects, it is possible to have better asthma control and an improved quality of life (QoL) in children and their families. Real-life studies demonstrated the steroid-sparing effect and a smaller number of exacerbations in omalizumab-treated children. In particular, the ANCHORS study (Asthma in Children: Omalizumab in Real Life in Spain) demonstrated, since the first year of treatment, a significant decrease in the exacerbation rate, steroids, and FeNO [21]. In addition, the use of omalizumab in pediatric patients is associated with a decrease in seasonal exacerbations induced by respiratory viruses [20,22,23,24]. This finding could be explained by the ability of omalizumab to restore the IFN response against viral infections (rhinovirus and influenza), preventing exacerbations [17].

A randomized controlled trial conducted by Sheehan et al. [25] demonstrated that omalizumab is more effective in children with increased levels of aeroallergen sensitizations, total serum IgE, and total serum eosinophil. Indeed, potential predictors of good asthma response to omalizumab treatment are represented by biomarkers such as peripheral eosinophil counts (>260/μL), fractional exhaled nitric oxide (FeNO) (>20 ppb), the presence of allergen-driven symptoms and childhood-onset asthma [14]. On the contrary, children older than 12 years with exacerbations within the last 6 months, a forced expiratory volume in 1 s (FEV_1_) < 90% of the predicted value, or comorbidities (obesity, gastroesophageal reflux, chronic rhinosinusitis, nasal polyps, and psychological disorders) have a greater risk of poor response to treatment [17]. Boek et al. conducted a multicenter, placebo-controlled, three-arm, randomized, parallel-group study on 52 patients aged >18 years, with house dust mite (HDM)-driven asthma. The study compared the efficacy of allergy immunotherapy (AIT) in patients treated with and without omalizumab. The results showed that the combination of AIT and omalizumab is more effective in improving the control of asthma symptoms and in reducing the daily dose of ICS compared to the use of omalizumab or AIT alone, but further studies are needed to confirm these findings [26].

The duration of the therapy with omalizumab is not well defined, but at least a 4-month treatment is recommended [14].

An observational 6-year study conducted by Nieto Garcia et al. has demonstrated that a beneficial effect can be maintained long term, with a good safety profile [21]. New data are emerging regarding the discontinuation of the therapy with omalizumab. A recent prospective cohort study [27] showed a reassuring clinical and functional effect of omalizumab for at least 1 year after discontinuation in children and adolescents with severe asthma without an increase in asthma exacerbations or worsening of the symptoms. To assess asthma control, the study relied on the Global Initiative for Asthma (GINA), the Composite Asthma Severity Index (CASI), spirometry, and the Pediatric Asthma Quality of Life Questionnaire (PAQLQ). Therefore, omalizumab discontinuation, after good disease control, could be a safe option in most children with severe asthma, even if it is important to continue to monitor children closely, as data are still limited.

### Safety

Omalizumab is generally tolerated in children and adolescents. A recent prospective multicentric surveillance study was conducted by Nakamura et al., demonstrating that adverse and serious events occurred in 47.2% and 23.6% of patients, respectively. Pyrexia (2.4%) and urticaria (1.6%) were the most frequent adverse drug reactions [28]. Anaphylaxis was demonstrated only in 0.2% of patients [29].

## 6. Mepolizumab

Mepolizumab is an anti-IL 5 monoclonal antibody, currently approved for children aged ≥6 years. It is administered via subcutaneous injection every 4 weeks. The dose is 100 mg for children aged ≥12 (>40 Kg) and 40 mg for children aged 6–11 (<40 Kg) [30].

Its mechanism is related to the binding of circulating IL-5, making it possible to prevent the interaction with its receptors and finally leading to the reduced production and survival of eosinophils [16].

Mepolizumab is recommended as an additional therapy for children affected by severe asthma with a high blood eosinophils count (≥150/μL) in the absence of steroid treatment and a history of frequent asthma exacerbations [14,30]. Another selection criterion for using mepolizumab in pediatrics is the presence of nasal polyps as an asthma comorbidity [31]. Its efficacy has also been proved for eosinophilic granulomatosis with polyangiitis (EGPA), hypereosinophilic syndrome or chronic rhinosinusitis with nasal polyposis [14]. Deepening the efficacy of mepolizumab, some studies demonstrated a significant reduction in severe asthma exacerbation, use of OCS and hospitalization in patients affected by severe asthma. A significant improvement in symptoms, FEV_1_, and quality of life (QoL) was also shown [32,33]. The efficacy of mepolizumab was also demonstrated in pediatric age in all fields shown in adults [20,34,35,36]. Indeed, the results of a randomized, double-blind, placebo-controlled, parallel-group study [37] have been recently published. The study was conducted across nine urban medical centers in the United States, recruiting 290 children and adolescents with asthma prone to eosinophilic exacerbation living in socioeconomically disadvantaged neighborhoods. Compared to guideline-based care alone, the study demonstrated an improvement in asthma exacerbations over 52 weeks in patients treated with mepolizumab. However, no significant differences were found in secondary outcomes, including time to the first exacerbation, lung function, or Composite Asthma Severity Index (CASI). An important finding was that the response to mepolizumab correlated with the pre-treatment expression of specific airway inflammatory pathways. Through transcriptome profiling techniques, it was possible to identify the inflammatory pathways that contribute to exacerbations despite the reduction in eosinophil-related inflammation. A greater baseline expression of inflammatory pathways involving T2 inflammation and eicosanoid metabolism and eosinophils observed a risk for greater flares in the placebo group and better response to treatment with mepolizumab. However, components of eosinophil activation associated with airway mucus hypersecretion persisted despite mepolizumab therapy and were associated with an ongoing risk of exacerbation. Therefore, mepolizumab contributed to reducing eosinophil count and T2 inflammation, but refractory mechanisms of eosinophils and mucins regulating the epithelium contribute to the risk of exacerbation and incomplete responses to mepolizumab. Furthermore, an elevated baseline expression of multiple non-T2 inflammatory pathways from the epithelium observed the risk of flares in the mepolizumab group, and the expression of many of these pathways increased during mepolizumab therapy. Additionally, the limited therapeutic response observed in this population could be partially explained by adverse environmental exposures of urban children that drive these epithelial inflammatory pathways. The duration of the therapy with mepolizumab is not well defined. Regardless, at least a 4-month treatment is recommended [14]. It is known that the continuous administration of mepolizumab makes it possible to maintain a positive therapeutic effect [38]. In addition, the discontinuation of mepolizumab can cause a relapse after 3–6 months, showing a decrease in asthma control and high peripheral eosinophilia [32]. Potential predictors of good asthma response to mepolizumab treatment in pediatric age are high peripheral eosinophil counts, a high number of exacerbations in the previous year, adult-onset asthma, nasal polyposis, maintenance OCS at baseline and low lung function (FEV_1_ < 65%) [14,39]. On the contrary, the effectiveness of mepolizumab does not appear to be related to IgE levels and the presence of atopy [40].

### Safety

Mepolizumab is generally tolerated in children and adolescents. In children, the adverse effects are related to the skin, subcutaneous tissue, and the nervous system (e.g., headache, syncope, dizziness) [14]. An open-label, uncontrolled, repeat-dose extension study was conducted by Gupta et al., demonstrating that adverse events and serious adverse events occurred in 90% and 27% of patients, respectively, in pediatric age [34]. Anaphylaxis was rare. Generally, there is a positive benefit–risk profile for mepolizumab in children with severe asthma with an eosinophilic phenotype [34].

## 7. Benralizumab

Benralizumab is an anti-IL 5Rα monoclonal antibody, approved for children aged ≥12 years and adults affected by severe eosinophilic asthma (serum eosinophils ≥300/μL) [14,17].

It is administered via subcutaneous injection with a pre-filled autoinjector syringe every 4 weeks for the first three doses and every 8 weeks afterwards, allowing for the least frequent administration schedule amongst the biologics now available for severe asthma. The dose is 30 mg [41].

The target is the alpha subunit of the IL-5 receptor expressed on eosinophils and basophils, causing the inhibition of their activation and the rapid and complete depletion of eosinophils by cytotoxicity mediated by natural killer cells [40,42,43].

Regarding the efficacy of benralizumab, some placebo-controlled phase 3 studies have been conducted involving adults and adolescents from 12 years of age [44,45,46,47]. A significant reduction in severe asthma exacerbation and the use of OCS and a significant improvement in symptoms and FEV_1_ were demonstrated in patients affected by severe asthma [16,20,44,45,47,48]. On the other hand, no randomized controlled trials involving children younger than 12 years of age are available. Just et al. reported the efficacy of benralizumab in a case series involving six children from 5 to 10 years [49]. Predictors of response appear to be a high baseline exacerbation rate, higher blood eosinophils, nasal polyposis, low baseline FEV_1_, and dependence on OCS [40].

The duration of therapy with benralizumab is not well defined, but at least 4 months of therapy is suggested [14].

### Safety

Benralizumab is generally well tolerated [46,50,51]. Specifically, frequent adverse events include injection site reactions and nasopharyngitis [40]; anaphylaxis is rare [48]. Concerns have been raised since the complete block-out of the eosinophils that play an important role in innate immunity. However, no apparent association exists between treatment and the increased risk of infections or malignancies [52].

## 8. Dupilumab 

Dupilumab is an anti-IL 4Rα monoclonal antibody, currently approved for children aged ≥6 years with severe type 2 asthma [14,53].

It is administered subcutaneously every 2 weeks. In adolescents from 12 years of age, the first dose is 600 mg, with the next doses of 300 mg every two weeks for those with OCS-dependent asthma or patients with severe asthma and co-morbid moderate-to-severe atopic dermatitis, while in other cases, the dose is 400 mg followed by 200 mg every two weeks [54]. In children affected by severe asthma (6–11 years of age), the dose and frequency are based on weight [14,17,54]: 16–30 kg: 100 mg every two weeks or 300 mg every four weeks; 31–60 kg: 200 mg every two weeks or 300 mg every four weeks; and >60 kg: 200 mg every 2 weeks. Dupilumab’s target is the IL-4α receptor, altering the inflammatory signal. Previously, in studies involving adolescents from 12 years of age and adults affected by severe asthma, dupilumab showed a significant reduction in severe asthma exacerbation, OCS administration and hospitalization, with a significant improvement in symptoms and FEV_1_ [55,56,57]. Dupilumab was recently approved in asthmatic children above 6 years of age based on the phase 3 VOYAGE study [53]. The post hoc analysis of VOYAGE [58] evaluated its efficacy in patients aged 6–11 years with type 2 asthma independently from the evidence of allergy-driven asthma (serum total IgE ≥ 30 IU/mL and ≥1 perennial aeroallergen-specific IgE ≥ 0.35 kU/L). Surprisingly, the annual severe exacerbation rates were significantly curbed in both groups when compared to the placebo, while children with allergic asthma presented additionally an improvement in percent-predicted pre-BD FEV_1_, pre-bronchodilator FEV_1_, and asthma control evaluated through the Asthma Control Score (ACQ)-7. Values of FeNO > 50 ppb and eosinophils > 300/μL are associated with a better response [17]. Other selection criteria for using dupilumab in pediatric asthma are atopic dermatitis and chronic rhinosinusitis with nasal polyps (CRSwNP) [59,60]. Especially in the case of atopic dermatitis, dupilumab can be chosen as the first-line therapy over other biologics due to the efficacy of dupilumab in the reduction in objective signs and symptoms related to atopic dermatitis, the QoL score, and depression and anxiety score [40]. Additionally, dupilumab has been approved for atopic dermatitis from 6 months of age and for eosinophilic esophagitis in adolescents from 12 years of age, while in adults, it is also approved for CRSwNP and prurigo nodularis [54].

The duration of the therapy with dupilumab is not well defined, but at least a 4-month treatment is recommended [14].

### Safety

Dupilumab is generally well tolerated in adults, but the evidence of its safety in pediatric age is still limited due to the duration of the available studies [61]. The open-label extension study EXCURSION, conducted on 365 children affected by moderate and severe asthma enrolled from the VOYAGE study [62], confirmed the safety profile and the proportion of adverse events to treatment. Specifically, the most common adverse events were upper respiratory tract infections such as nasopharyngitis and pharyngitis [62]. Anaphylaxis is rare [13,56,63]. Eosinophilic granulomatosis with polyangiitis is reported in adulthood but not in children [64]. Transient hypereosinophilia with no clinical symptoms has been reported, raising concerns about additional potential side effects to other organs [61].

## 9. Tezepelumab 

Tezepelumab is an IgG2λ monoclonal antibody directed to the circulating thymic stromal lymphopoietin (TSPL), a cytokine involved in the pathway of T2 inflammation, preventing its receptor binding. Through this, tezepelumab can block the inflammatory cascade at a very high level [14,65]. Indeed, TSLP is an upstream communicator between airway epithelium and immune cells in reaction to allergens, microbes, and pollutants. Through its blocking ability, tezepelumab operates in multiple fronts, mitigating local inflammation without taking into account the blood eosinophil level, interfering with T2 and T17 responses through dendritic cells, and interacting with mast cells and airway smooth muscle cells [66].

In 2022, tezepelumab was approved in adults and adolescents affected by severe asthma from 12 years of age regardless of the asthma phenotype, making it the only biologic approved with no biomarker limitation [65].

The drug is administered subcutaneously; the dose is 210 mg every 4 weeks [17]. The NAVIGATOR trial, a multicenter phase 3, randomized, double-blind, placebo-controlled trial, evaluated the efficacy of tezepelumab involving patients from 12 to 80 years of age (82 adolescents) [66], including patients without an eosinophilic phenotype (blood eosinophils less than 300/μL). Tezepelumab showed a statistically significant improvement in FEV_1_ and QoL and reduced severe exacerbations, regardless of the eosinophil count. However, it should be mentioned that this trial, when restricted to the adolescent participants, failed to show a significant reduction in exacerbation frequency (RR 0.7; 95% CI 0.34–1.46), possibly due to the statistical power. Nevertheless, its use has been approved in adolescents, but further data assessing its efficacy in this age group are needed [67]. Tezepelumab was also found to lower blood eosinophil count and levels of FeNO and IgE [66].

### Safety

In the DESTINATION trial [68], tezepelumab was found to be well tolerated for up to two years, but the adolescent population was not considered separately from adults. Common colds, infections of the sinuses, throat and airways, headaches, worsening of asthma, and airway inflammation were reported as adverse events [69].

## 10. A Tailored Biologic Therapy for Each Pediatric-Specific Severe Asthma

**Figure 1:**
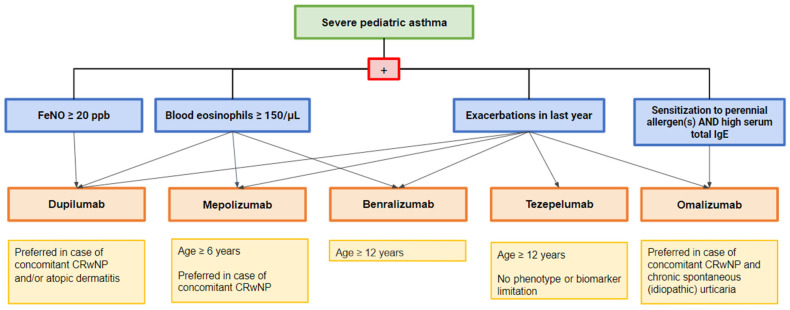
Therapeutic algorithm to guide the choice of biologics in children affected by severe asthma.

When a child presents with uncontrolled asthma symptoms, the first step is collecting a detailed history and a focused approach to assessing indirect symptoms of atopy, social condition, and the presence of comorbidities and environmental factors, such as sensitization and exposure to allergens. The next step is a careful examination to search for signs attributable to asthma [8]. It is also mandatory to check the diagnosis of asthma since up to 12–50% of individuals with a diagnosis of severe asthma have been wrongly attributed to asthma [70]. Spirometry and an evaluation of a bronchodilatation response are needed to assess pulmonary function at baseline and obtain objective evidence of variable limitation to expiratory airflow, confirmed by an FEV_1_ change > 10% of the predicted value [71]. As the diagnosis of asthma is confirmed, an optimized treatment must be undertaken. Subsequently, a re-evaluation should be performed every 3–6 months, focusing both on all comorbidities and modifiable factors, such as poor adherence, exposure to allergens, passive or active smoking, vaping, and psychosocial factors that can lead to poor symptom control and eventually taking into consideration other differential diagnoses. After having evaluated the patient for at least three months [61], if asthma remains uncontrolled despite adequate treatment with a high dose of ICS and long-acting beta2-agonist or the patient requires a high dose of ICS-LABA to control asthma, having excluded comorbidities and risk factors, the diagnosis of severe asthma is then confirmed [14]. Based on these findings, children with severe asthma should undergo airway phenotyping for an individualized management plan [8]. The determination of the airway phenotype involves assessing inflammatory pathways through serum IgE, serum eosinophils, FeNO, induced sputum, bronchoalveolar lavage, or bronchial biopsy [49]. Combining serum and respiratory biomarkers can define the patient’s endotype, allowing one to determine the most suitable treatment at an individual level [49]. This is also possible in pediatric age because several biologics have been approved, such as omalizumab, mepolizumab, dupilumab, benralizumab, and tezepelumab. In the complex choice of the most suitable biological treatment for the patient, clinicians should take into account many factors such as the indication of the biologic, the phenotype, the presence of predictors of treatment response, the local eligibility, the cost, the frequency, and the route of administration, and the patient and caregiver preference and compliance. Starting from the recommendations and evidence on the prediction of good asthma response to the treatment, it is reasonable to propose a therapeutic algorithm to choose a tailored biological therapy for each specific phenotype of childhood asthma. Specifically, the presence of aeroallergen sensitization and high eosinophil blood counts can suggest the use of omalizumab and dupilumab. In this context, when the level of serum total IgE is high, omalizumab can represent the best choice. When FeNO is high, dupilumab can be chosen. In case of no-elevated T2 biomarkers, tezepelumab can instead be considered [14]. A high eosinophil blood count without aeroallergen sensitization can encourage the use of mepolizumab and benralizumab. Finally, the possible presence of comorbidities can also influence the correct prescription of biologics. For example, omalizumab and dupilumab may be considered when atopic dermatitis is present, while mepolizumab and dupilumab may be considered in the case of CRSwNP (Figure 1).

## 11. Evolution of Childhood Asthma after Prolonged Biological Treatment

Thanks to the evolution in asthma therapy, we are increasingly moving towards the concept of remission of disease. Even though there is no consensus in the literature on the definition, generally, it refers to reaching disease control, that is, a condition characterized by the absence of symptoms and exacerbations, with normal lung function, with or without therapy for at least one year. According to a Delphi Consensus, partial clinical remission is defined by the absence of the need for OCS and by the presence of two of the three criteria among the absence of symptoms, the absence of exacerbations and stability of the respiratory function [72]. Remission is distinguished from complete recovery, which refers to the absence of the characteristic airway structural changes that occur in long-standing asthma, such as epithelial hyperplasia and metaplasia, changes in mucus-secreting cells, thickening of submucosal tissue, subepithelial fibrosis, muscle cell hyperplasia and angiogenesis, which overall lead to airway remodeling with a progressive, irreversible loss of lung function. For this reason, even patients in remission can have exacerbations due to chronic structural changes in the airways [73]. Favorable factors for remission include mild disease, preserved lung function, good asthma control, young age, early onset asthma, short duration of disease, mild bronchial hyperresponsiveness, few comorbidities, and smoking cessation or no smoking. However, patients in advanced stages of severe asthma are unlikely to achieve remission [74]. Currently, evidence on the potential effect of the current therapies on reversing airway remodeling is scarce. The available studies have been conducted only in adults and in small size samples, showing a potential in vivo effect in airway remodeling evaluated through CT (omalizumab [75,76,77] and mepolizumab [78]) with a reduction in airway wall thickness and wall area, and increase in the tracheal lumen area, and through bronchial biopsies (omalizumab [79,80,81] and benralizumab [82]) with a reduction in reticular basement membrane thickness, in airway smooth muscle proteins, and in fibronectin deposit in airway submucosa. To date, no studies are available on the in vivo effects of dupilumab on airway remodeling [83]. Furthermore, even after discontinuation, biologic therapies possibly induce better control of asthma, thanks to their effect on airway remodeling. A study conducted by Jeffery et al. [84] on a cohort of 4960 asthmatics showed that 10.2% of the patients who discontinued biologic treatment for at least 6 months and 9.5% of those on biologic treatment had a 50% or more increase in asthma exacerbations, demonstrating that asthma exacerbations in patients who discontinued biologics had similar rates to controls who continued therapy. However, the study lacks a separate analysis for the pediatric age cohort. Recently, the results of a large-scale study on omalizumab have been published [85]. Data from 16,750 adults and 2453 children treated with omalizumab for at least 16 weeks were analyzed, with the follow-up continued for 10 years after the discontinuation of the therapy. While the control of asthma was reached respectively in 70%, 39%, and 24% of adults 1, 2, and 3 years after the discontinuation of omalizumab, higher proportions were observed in children (76%, 44%, and 33%, respectively). Noteworthy, healthcare costs were diminished in the long term for both adults and children, thanks to a reduced use of OCS and rate of hospitalizations. Ultimately, it is important to implement research into new therapies capable of reversing the remodeling process and restoring lung function. Studies on new biological drugs that have clinical remission as their primary aim are desirable, with a focus on halting disease progression, potentially reversing the damage already caused to the airways [73], especially in pediatric age.

## 12. Conclusions

It is fascinating how asthma treatment in the pediatric age has evolved since the introduction in 2005 of the first biologic drug, omalizumab. In the last 18 years, four other biological drugs (mepolizumab, benralizumab, dupilumab and tezepelumab) have been approved for pediatric age. Biologics have the potential to dramatically change the evolution of asthma, based on the physiopathological mechanisms of this illness [49], although it is challenging to identify the right drug for the right patient. The comparison of biologics is necessary to target treatment decisions and reduce related healthcare costs [8] to guide a shared and validated therapeutic algorithm currently lacking in the pediatric age. Moreover, the duration and the indications for discontinuing biological therapies are still debated as their possible role in asthma prevention and as disease modifiers are not elucidated [86]. Further studies are required to address the many unresolved issues in this field of research, allowing a more precise patient-targeted therapy in childhood asthma, potentially interfering with its natural history.

## Figures and Tables

**Table 1:** Evolution of asthma treatment over time.

Year	Main Advancement of Knowledge Related to Asthma
1754 BCE	The first finding of a pathology that recalled asthma in the Code of Hammurabi
460–370 BCE	Invention of the term “asthma” (from the Greek “azein”) by Hippocrates from Kos
XVIII century	Development of the inhaler by John Mudge
1967	Development of inhaled albuterol
1972	Development of inhaled beclomethasone
1995	First edition of GINA * recommendations
2000s	The concept of “asthma control” became the main guide in asthma treatment
2005	Introduction of biologics (omalizumab) as targeted therapy in asthma in Europe
2015	Approval of mepolizumab for severe eosinophilic asthma
2017	Approval of benralizumab for severe eosinophilic asthma
2019	Approval of dupilumab for severe asthma with type 2 inflammation
2022	Approval of tezepelumab for inadequately controlled severe asthma

* GINA: Global Initiative for Asthma.

**Table 2:** Biologics currently available for severe asthma in pediatric age with the relative approved age of use, molecular target, and dosage.

Biological Drug	Molecular Target	Approved Age for Use	Indication	Administration Dose for Asthma
Omalizumab	IgE	≥6 years	Severe allergic asthma	0.016 mg/kg/IgE (I.U./mL) every 2–4 w°
Mepolizumab	IL5	≥6 years	Severe eosinophilic asthma	6–11 y#, <40 Kg: 40 mg every 4 w >12 y, >40 Kg: 100 mg every 4 w
Benralizumab	IL 5Rα	≥12 years (USA)	Severe eosinophilic asthma	30 mg every 4 w for the first three doses, every 8 w afterwards
Dupilumab	IL 4Rα	≥6 years	Severe T2-high asthma	6–11 y, 15–30 kg: 100 mg every 2 weeks or 300 mg every 4 w 6–11 y, 31–60 kg: 200 mg every 2 w or 300 mg every 4 w >12 y, >60 kg: 200 mg every 2 w
Tezepelumab	TSLP *	≥12 years	Severe asthma	210 mg every 4 w

* Anti-circulating thymic stromal lymphopoietin, y#: years old, w°: weeks.

## Data Availability

No new data were created or analyzed in this study. Data sharing is not applicable to this article.
